# Tetramethylpyrazine inhibits CTGF and Smad2/3 expression and proliferation of hepatic stellate cells

**DOI:** 10.1080/13102818.2014.984382

**Published:** 2015-01-28

**Authors:** Jun Li, Ni Dong, Shuang Cheng, Xiaosheng Li, Wenli Wang, Ying Xiang

**Affiliations:** ^a^ Department of Endodontics, The Affiliated Hospital of Stomatology, Chongqing Medical University, Chongqing400010, China; ^b^ Chongqing Key Laboratory for Oral Diseases and Biomedical Sciences, Chongqing401147, China; ^c^ Department of Gastroenterology, The Second Affiliated Hospital of Chongqing Medical University, Chongqing400010, China

**Keywords:** tetramethylpyrazine, TGF-β1, connective tissue growth factor, Smad, hepatic stellate cells

## Abstract

To study the effects of tetramethylpyrazine (TMP) on the proliferation of hepatic stellate cells-T6 (HSC-T6), and the expression of connective tissue growth factor (CTGF) and Smad2/3 in these cells, HSC-T6 cells were cultured with TMP at different concentrations after transforming growth factor-β1 (TGF-β1) stimulation. MTT assay was used to assess the cell proliferation. Cells were divided into the control group, TGF-β1-treated group and TMP-treated groups, which were treated with different concentrations of TMP. Immunocytochemistry and western blot were performed to detect the expression levels of CTGF and Smad2/3 in HSC-T6 cells. MTT analysis indicated that TMP significantly inhibited the proliferation of HSC-T6 cells, in dose-dependent and time-dependent manners. Immunocytochemistry detection and western blot showed that TMP could diminish TGF-β1-induced CTGF over-expression in HSC-T6 cells. Similarly, the enhancing effects of TGF-β1 on Smad2/3 expressions in HSC-T6 cells could also be counteracted by TMP treatment. Nuclear translocation of Smad2/3 was blocked by TMP treatment. Correlation analysis suggested a positive correlation between CTGF and Smad2/3 expression levels in HSC-T6 cells. TMP exerts anti-hepatic fibrosis effect through decreasing the expression of CTGF and Smad2/3, as well as inhibiting the proliferation of HSC-T6 cells. Our study provides cellular and molecular bases for further application of TMP in the clinical treatment for hepatic fibrosis.

## Introduction

Hepatic fibrosis is a common cellular wound-healing response to chronic tissue injury, which is characterized by proliferation and migration of hepatic stellate cells (HSCs) producing collagen and extracellular matrix (ECM). Several signalling pathways and molecules have been found to participate in hepatic fibrosis. Transforming growth factor-β1 (TGF-β1) is one of the most important fibrogenic cytokines,[1] and TGF-β1-Smad signalling pathway is the major pathway involved in hepatic fibrosis,[2] in which Smad2/3 works as an intermediate step to specifically transduce signals from TGF-β.[3] On the other hand, connective tissue growth factor (CTGF) is a newly discovered cytokine playing a key role in the pathogenesis of tissue and organ fibrosis. CTGF has been shown to promote the activation, synthesis and secretion of ECM in glomerular mesangial cells and HSCs.[4–6]

Tetramethylpyrazine (TMP) is one of the active ingredients of the Chinese herb *Ligusticum wallichii* Franchat (*L. wallichii*; Chuan Xiong), which has been widely used to treat hypertension and cardiovascular diseases. As an alkaloid, TMP exerts versatile biological functions, such as expanding blood vessels, inhibiting platelet aggregation and other antioxidant and immune-regulating effects.[7–9] In recent years, clinical and experimental studies have indicated that TMP could protect hepatocytes in hepatic injuries and reduce liver fibrosis.[10,11] Our recent study also suggests that TMP significantly reduces serum levels of type I and III collagen and the degree of liver fibrosis in rat models. Furthermore, TMP also inhibits the expression of TGF-β1, CTGF and a-smooth muscle actin, with particularly pronounced inhibition of CTGF.[12]

In this study, we investigated the effects of TMP on the proliferation of HSC-T6 cells, as well as the expression of CTGF and Smad2/3 in these cells. The cellular and molecular mechanism(s) through which TMP prevented liver fibrosis were also investigated and discussed.

## Materials and methods

### Cell line and reagents

Hepatic stellate cells, HSC-T6, were obtained from the Institute of Cancer, Chinese Academy of Medical Sciences, Beijing, China. These were HSC from SD rats, transfected with SV40, which exhibited activated phenotypes. RPMI 1640 cell culture medium was from Hyclone, Logan, UT, USA. Fetal bovine serum (FBS) was purchased from Zhejiang Tianhang Biological Technology Co. Ltd., Hangzhou, Zhejiang, China. Recombinant human TGF-β1 (No. 100-21C) was purchased from Peprotech Ltd., London, UK. TMP was purchased from Medisan Pharmaceutical Co. Ltd., Harbin, Heilongjiang, China. Rabbit anti-mouse anti-CTGF (H-55) polyclonal antibody and rabbit anti-Smad2/3 polyclonal antibody were purchased from Santa Cruz Biotechnology, Santa Cruz, CA, USA. Protein extraction kits and polyvinylidene difluoride (PVDF) films were purchased from Bio-Rad Laboratories, Hercules, CA, USA. ECL chemiluminescence reagents were purchased from Amersham Biosciences, Piscataway, NJ, USA. SP Kits for immunohistochemical detection and diaminobenzidine (DAB) chromogenic reagent kits were purchased from ZSGB-SIO, Beijing, China. dimethyl sulphoxide (DMSO) and MTT (3-(4,5-dimethylthiazol-2-yl)-2,5-diphenyltetrazolium bromide) were purchased from Sigma, St. Louis, MO, USA.

### Cell culture and drug treatment

HSC-T6 cells were cultured in RMPI 1640 medium, supplemented with 10% FBS, antibiotics (100 units/mL penicillin and streptomycin each) and 4 mmol/L glutamine, in a 37°, 5% CO_2_ incubator with saturated humidity. When growing to 80%–90% confluence, the cells were ready for passaging. Cells from 3–5 passages were used for subsequent experiments.

In the TGF-β1-treated group, cells were only treated with 5 ng/mL TGF-β1. In the TMP-treated groups, cells were first treated with 5 ng/mL TGF-β1 for 30 min,[6] and then incubated with TMP at the concentrations of 50, 100, 200, 400, 600 and 1000 μg/mL, for a further 24, 48 and 72 h, respectively. Cells without treatments were used as blank control. Following drug treatments, these cells were subjected to various measurements.

### MTT proliferation assay

HSC-T6 cells were seeded onto a 96-well plate at the density of l × 10^5^ cells/mL. After drug incubation, culture medium was exchanged by 200 μL serum-free RPMI 1640 medium containing 1 mg/mL MTT reagent, and the plate was incubated at 37 °C for 4 h. Then, culture supernatant was discarded, and 200 μL DMSO was added into each well, oscillating for 10 min until completely dissolved. The optical density (OD) at 570 nm was read in a micro-plate reader. Since MTT could be converted to formazan only by mitochondria in viable cells, cell proliferation was determined by comparing control and incubated cells, and the inhibition ratio (IR) was calculated as follows:

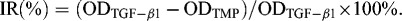



### Immunocytochemistry

HSC-T6 cells were seeded onto a 24-well plate. After 48 h incubation with TGF-β1 or TMP (100, 200, 400, and 600 μg/mL), immunocytochemistry was performed with the SP Kits and the DAB chromogenic reagent kits, according to the manufacturers’ instructions. Images taken by a digital camera were analysed with CMIAS2000 image analysis system (Beijing University of Aeronautics & Astronautics, Beijing, China). Brown granules were considered as positive staining, six fields were randomly selected from each well and the average optical density was used for statistical analysis.

### Western blot analysis

After drug treatments, cells were lysed with protein extraction kits. Protein samples were subjected to SDS-PAGE, and then electro-transferred onto a PVDF film. After blocking with 5% non-fat milk in TBST (10 mM Tris-HCl, 100 mM NaCl, 0.1% Tween-20, pH 7.5), the film was incubated with rabbit anti-mouse anti-CTGF (H-55) polyclonal antibody (1:100 dilution) or rabbit anti-Smad2/3 polyclonal antibody (1:100 dilution) in TBST at 4 °C overnight. Then, secondary antibody (1:800 dilution) in TBST was used for incubation at room temperature for 90 min. The blots were developed using ECL chemiluminescence reagents. Quantity One image analysis system (BioRad) was used to analyse the optical density of the protein bands. Relative levels of target proteins were determined as compared to internal reference proteins.

### Statistical analysis

Data were expressed as mean ± SD. SPSS 15.0 software was applied to perform analysis of variance (ANOVA) and paired *t*-tests. Spearman rank-order correlation was employed for correlation analysis. *P* < 0.05 was considered statistically significant.

## Results and discussion

### TMP inhibits the proliferation of HSC-T6 cells

**Figure 1.**
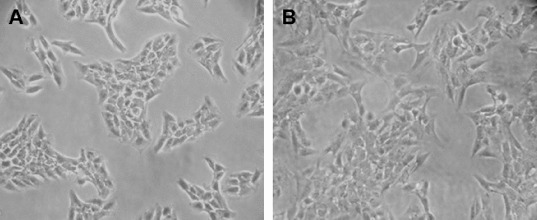
HSC-T6 cell cultures. HSC-T6 cells were cultured with RMPI 1640 medium for 24 h (A) and 48 h (B) (×200).

**Figure 2.**
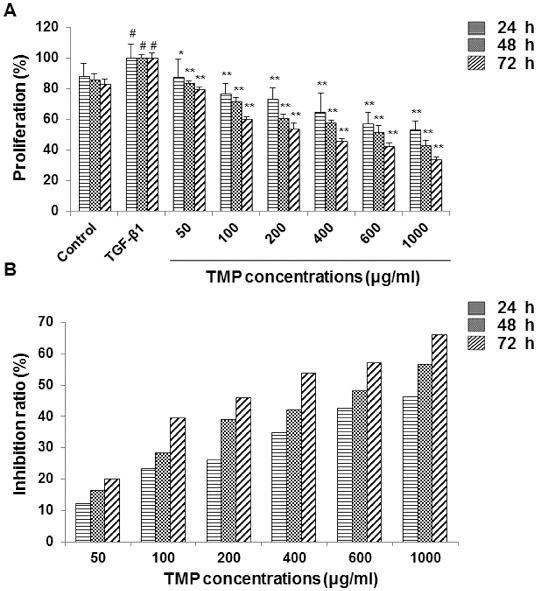
Effects of TMP on the proliferation of HSC-T6 cells. (A) HSC-T6 cell proliferation was measured with MTT assay. In the control group, cells received no drug treatment; in the TGF-β1-treated group, cells were treated with 5 ng/mL TGF-β1; in the TMP-treated groups, cells were first incubated with 5 ng/mL TGF-β1 for 30 min, and then treated TMP at concentrations of 50, 100, 200, 400, 600 and 1000 μg/mL for 24, 48 and 72 h, respectively. (B) Inhibition ratios of TMP on HSC-T6 cell proliferation. *n* = 6; compared with the blank control, ^#^ *P* < 0.05; compared with the TGF-β1-treated group, *P < 0.05, ^**^ *P* < 0.01.

HSC-T6 cells adhered to the surface at 24 h after passaging, with cell body enlarged and polygonal appearances. Cells were surrounded with elongated processes, and exhibited the typical myofibroblast-like morphology (Figure 1(A)). After another 24 h, cells grew into clusters, with thickened processes and close connections (Figure 1(B)). We investigated the influence of TMP on the proliferation of HSC-T6 cells. Cells were first incubated with 5 ng/mL TGF-β1 for 30 min, and then treated with TMP at indicated concentrations (100, 200, 400, 600 and 1000 μg/mL) for a further 24, 48 and 72 h, respectively. MTT assay was performed to assess cell proliferation. Our results indicated that TGF-β1 treatment alone significantly stimulated the proliferation of HSC-T6 cells (*P* < 0.05). In the TMP-treated groups, even though these cells were pre-treated with TGF-β1, the following TMP administration dramatically reduced the cell proliferation, at all concentrations tested (100–1000 μg/mL) (*P* < 0.05 and *P* < 0.01; Figure 2(A)). The IR was elevated along with the increasing treatment concentrations of TMP (*P* < 0.05; Figure 2(B)). When the incubation periods were prolonged (48 or 72 h), IRs were elevated by TMP treatment to a much greater extent (Figure 2(B)). Furthermore, correlation analyses revealed that there was an obvious linear relationship between IRs and TMP concentrations (for 24-h treatment, *r* = 0.955, *P* < 0.01; for 48-h treatment, *r* = 0.962, *P* < 0.01; for 72-h treatment, *r* = 0.976, *P* < 0.01). These results suggest that TMP significantly inhibits the proliferation of HSC-T6 cells, in dose-dependent and time-dependent manners.

### TMP decreases TGF-β1-induced CTGF over-expression in HSC-T6 cells

**Figure 3.**
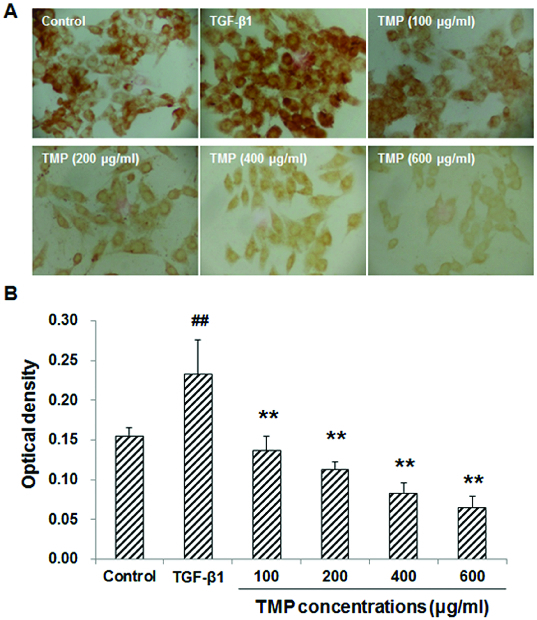
Effects of TMP on TGF-β1-induced CTGF expression in HSC-T6 cells detected by immunocytochemistry. (A) Immunocytochemistry was performed to detect CTGF expression in HSC-T6 cells after drug administration (DAB staining, ×200). (B) Quantitative analysis of the average optical density of CTGF staining. *n* = 6; compared with the control group, ^##^ *P* < 0.01; compared with the TGF-β1-treated group, ^**^ *P* < 0.01.

**Figure 4.**
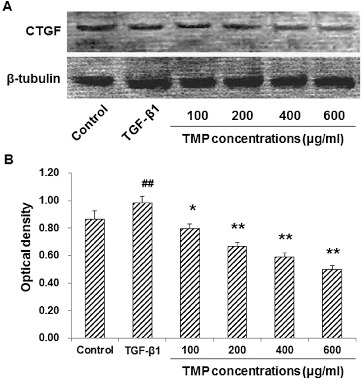
Effects of TMP on TGF-β1-induced CTGF expression in HSC-T6 cells detected by western blot. (A) Western blot was used to detect the protein expression levels of CTGF in HSC-T6 cells after drug administration. HSC-T6 cells were treated with nothing (blank control), 5 ng/mL TGF-β1 and TMP at concentrations of 100, 200, 400 and 600 μg/mL, respectively. (B) Quantitative analysis of the relative optical density of the protein bands. *n* = 6; compared with the blank control, ^##^ *P* < 0.01; compared with the TGF-β1-treated group, *P < 0.05, **P < 0.01.

When HSC-T6 cells are stimulated with TGF-β1, the expression levels of CTGF and Smad2/3 would be up-regulated in these cells. Subsequently, the production of ECM would also be enhanced to induce hepatic fibrosis.[13] We next tried to find out whether the TGF-β1-induced CTGF over-expression could be affected by TMP treatment. These cells were first incubated with 5 ng/mL TGF-β1 for 30 min, and then treated with TMP (100, 200, 400 and 600 μg/mL) for a further 48 h. Immunocytochemistry and western blot analysis were performed to detect the CTGF levels in these cells. Immunocytochemistry indicated that CTGF was mainly expressed in the cytoplasm. When treated with TGF-β1, HSC-T6 cells grew vigorously, with large cell bodies and extensive processes (Figure 3(A)). Compared with the blank control, the CTGF expression was significantly elevated after the TGF-β1 treatment (*P* < 0.01; Figure 3(B)). However, when further treated with TMP, these cells exhibited shrunk cell bodies and less-efficient processes. Furthermore, compared with the TGF-β1-treated group, the expression of CTGF was significantly decreased (Figure 3(A) and 3(B)). Moreover, western blot detection showed that the protein expression levels of CTGF declined when TMP concentrations increased, indicating that TMP significantly diminished CTGF expression (*P* < 0.05; Figure 4). Based on the above results, TGF-β1-induced CTGF over-expression could be decreased by TMP treatment in HSC-T6 cells.

### TMP decreases TGF-β1-induced Smad2/3 over-expression in HSC-T6 cells

**Figure 5.**
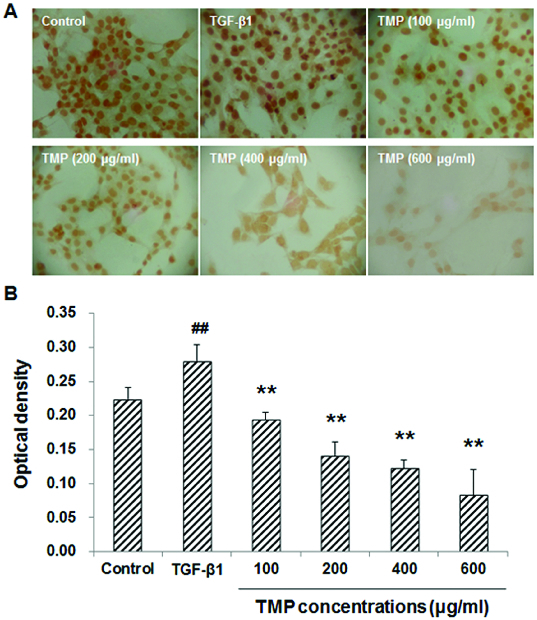
Effects of TMP on TGF-β1-induced Smad2/3 expression in HSC-T6 cells detected by immunocytochemistry. (A) Immunocytochemistry was performed to detect Smad2/3 expression in HSC-T6 cells after drug administration (DAB staining, ×200). (B) Quantitative analysis of the average optical density of Smad2/3 staining in the nuclei in HSC-T6 cells. *n* = 6; compared with the control group, ^##^ *P* < 0.01; compared with the TGF-β1-treated group, ^**^ *P* < 0.01.

**Figure 6.**
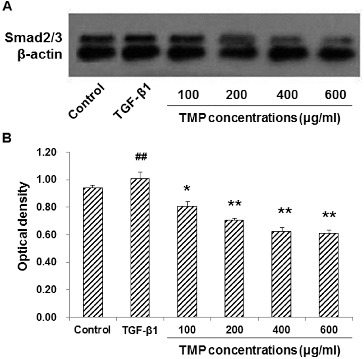
Effects of TMP on TGF-β1-induced Smad2/3 expression in HSC-T6 cells detected by western blot. (A) Western blot was used to detect the protein expression level of Smad2/3 in HSC-T6 cells after drug administration. HSC-T6 cells were treated with nothing (blank control), 5 ng/mL TGF-β1 and TMP at concentrations of 100, 200, 400 and 600 μg/mL, respectively. (B) Quantitative analysis of the relative optical density of the protein bands. *n* = 6; compared with the control group, ^##^ *P* < 0.01; compared with the TGF-β1-treated group, *P < 0.05, **P < 0.01.

To further investigate the influence of TMP on the TGF-β1-induced Smad2/3 expression in HSC-T6 cells, immunocytochemistry and western blot analysis were performed. Our results indicated that in the control group, Smad2/3 was mainly expressed in the cytoplasm, while in the TGF-β1-treated group, the majority of Smad2/3 was located in the nuclei, with less expression in the cytoplasm (Figure 5(A)). Quantitative analysis revealed that the TGF-β1 treatment dramatically increased the content of Smad2/3 within nuclei. However, following TMP treatments, the expression of Smad2/3 in the nuclei was significantly decreased in HSC-T6 cells (*P* < 0.01; Figure 5(B)). In line with this, western blot analysis suggested that the protein expression levels of Smad2/3 were significantly up-regulated in HSC-T6 cells treated with TGF-β1. When those cells were cultured further with TMP, the Smad2/3 protein expression levels declined significantly (*P* < 0.01; Figure 6). In addition, correlation analysis indicated that the expression levels of CTGF and Smad2/3 were positively correlated with each other (Spearman rank-order correlation coefficient *r* = 0.968, *P* = 0.007). These results suggest that the enhancing effects of TGF-β1 on Smad2/3 expression could be counteracted by the TMP treatment in HSC-T6 cells.

Hepatic fibrosis is characterized by the excessive accumulation and abnormal distribution of ECM in livers.[14] HSC activation is the cellular basis for hepatic fibrosis. TGF-β1 stimulation could change the phenotypes and functions of these cells, which synthesize and secrete large amount of ECM, and promote the formation and development of hepatic fibrosis.[15,16] Therefore, inhibition of the activation and proliferation of HSC has been an important target for the disease therapy. In the present study, using HSC-T6 cells, we explored the mechanism(s) through which TMP exerted protective effects against hepatic fibrosis and the involved cytokines. Our results indicated that TMP treatment, within a certain concentration range (100–1000 μg/mL), could significantly inhibit the proliferation of HSC, in dose-dependent and time-dependent manners. Longer incubation period and the higher concentrations applied led to a greater inhibition of cell proliferation. These results suggest that the anti-hepatic fibrosis activity of TMP might be attributed to the depression of HSC.

In the pathogenesis of hepatic fibrosis, TGF-β1-Smad signalling pathway plays an extremely important role in the proliferation, differentiation of HSCs as well as the synthesis of collagen,[17,18] in which Smad2/3 plays a major role. TGF-β1 binds to and activates its receptor, which mediates the phosphorylation of Smad2/3. Phosphorylated Smad2/3 interacts with Smad4 in the cytoplasm to form heterocomplexes, which translocate into the nuclei, regulating downstream target genes.[19,20] In this study, immunohistochemical staining showed that, in control HSC-T6 cells, Smad2/3 was mainly expressed in the cytoplasm. When stimulated with TGF-β1, the majority of Smad2/3 was observed in the nuclei, with less protein in the cytoplasm, indicating the translocation of Smad2/3. Moreover, expression of Smad2/3 within the nuclei was gradually decreased as TMP concentration increased. These results suggest that TMP declines the expression of Smad2/3, inhibits its nuclear translocation and blocks the signalling transduction process of TGF-β1.

CTGF is one of the most important cytokines in the pathogenesis of organ fibrosis.[21] In the process of TGF-β1 stimulation, the expression of CTGF is also elevated, which might be the central event in the activation of HSCs.[22,23] Recent studies have shown that TGF-β1 response elements in the CTGF promoter exist, which induce the gene expression and contribute to TGF-β1-related gene up-regulation. Our results showed that CTGF expression was positively correlated with TGF-β1 expression. Taken together, these data suggest that CTGF may be the downstream effector molecules in the TGF-β1 signalling pathway, exerting the pro-fibrotic effects.[24] Currently, Smads, Ras/MEK/ERK, tyrosine kinases and protein kinase C have been found to be involved in the CTGF signalling pathway.[25–27] For example, the coordination of the signalling pathways of Smads and Ras/MEK/ERK is needed to achieve the maximum stimulating effects of TGF-β1 on the secretion of CTGF.[28] Smads have also been found to be related with the basic expression of CTGF. The Smad-binding elements in the CTGF promoter could eliminate TGF-β1-induced CTGF expression and decrease the basic expression of CTGF. However, the exact relationship between CTGF and Smad2/3 has never been well established. In this study, our results indicated that TMP at certain concentrations significantly inhibited the expression of Smad2/3 and CTGF, which were positively correlated. Therefore, we postulate that CTGF would probably be regulated by the TGF-β1-Smad2/3 signalling pathway.

## Conclusions

In this study, our results showed that TMP decreased the expression of Smad2/3 and CTGF, and inhibited the proliferation of HSC-T6 cells, through which TMP would exert its anti-hepatic fibrosis effects. These findings provide cellular and molecular bases for further application of TMP in the clinical treatment for hepatic fibrosis.

## Disclosure statement

No potential conflict of interest was reported by the author(s).

## Funding

This work was supported by the Natural Science Foundation from Chongqing Science and Technology Committee [grant number 2006B135428]; the Research Foundation from Chongqing Municipal Health Bureau [grant number TCM [2005] 35].
